# Sublobar Resection for Early-Stage Lung Cancer: An Oncologically Valid Procedure?

**DOI:** 10.3390/jcm12072674

**Published:** 2023-04-03

**Authors:** Ian Diebels, Marc Dubois, Paul E. Y. Van Schil

**Affiliations:** 1 Department of Thoracic and Vascular Surgery, Heilig Hart Ziekenhuis, 2500 Lier, Belgium; 2 Department of Thoracic and Vascular Surgery, Antwerp University Hospital, 2650 Edegem, Belgium

**Keywords:** non-small-cell lung cancer (NSCLC), minimally invasive surgery, sublobar resection, segmentectomy, wedge excision, lobectomy

In the era of minimally invasive surgery, the role of sublobar resection comprising anatomical segmentectomy and wide wedge excision remains controversial. Its precise role from an oncological point of view still has to be exactly defined. Theoretically, a less invasive resection should lead to overall better post-operative respiratory function. However, until recently, hard evidence of survival equality or benefit over lobectomy was still lacking. Thus, lobectomy has been the preferred treatment of choice for most non-small cell lung cancer (NSCLC). Recently, two milestone trials have investigated the role of sublobar resection in the treatment of NSCLC.

The surgical goal of oncologic resections is achieving a microscopically complete resection (R0), which can be defined by free resection margins as per the latest, eighth edition of the tumour, node, metastasis (TNM) classification [1]. Correct staging is crucial as it determines treatment modalities and prognostic relevance [2]. Hence, it is concerning that local or regional recurrences still occur in R0 resected patients. Therefore, according to published guidelines, standard tumour resection and lymph node (LN) dissection are necessary to ensure true complete resection and correct evaluation in clinical trials. For this reason, the 2005 Complete Resection Subcommittee of the International Association for the Study of Lung Cancer (IASLC) proposed adding an additional stratification term, ‘uncertain resection R(un)’ [3]. R(un) is defined as a resection of a lung cancer with margins free of microscopic disease, but one of the following is present: incomplete LN dissection according to the standard systematic or lobe-specific criteria, metastasis in the highest resected mediastinal LN, carcinoma in situ confirmed at the bronchial resection margin, or positive cytology obtained during intraoperative pleural lavage [3]. The clinical significance of R(un) was confirmed by a re-analysis of 14,712 patients from the IASLC database, based on the previously mentioned criteria, whereby 57% of the R0 resections had to be reclassified to R(un). The R descriptor has an important impact on prognosis. In the conventional resection (R) status analysis, the 5-year overall survival (OS) was 73% for R0, which decreased to 36% for R1- and 28% for R2 resections. However, when the R0 dataset was reanalysed, now including R(un) with a positive highest resected LN, the 5-year OS for R0 resection was 55% while for R(un), it was only 45% [4]. The prognostic significance of the R factor is further analysed in other trials.

The first sublobar, landmark prospective randomised trial by Ginsberg et al., published in 1995, showed the inferiority of sublobar resection. The study included patients with a peripheral cT1N0 (<3 cm) lung cancer who were intraoperatively randomised between sublobar resections (n = 122, of which 82 were segmentectomies) and lobectomy (n = 125) [5]. Interestingly, 50% of NSCLC patients were excluded intraoperatively due to tumour size >3 cm, tumour location or configuration, or positive mediastinal LNs. A sublobar resection demonstrated a three-fold increase in the local recurrence rate (*p* = 0.008). A 30% increase in overall death rate (*p* = 0.08) and 50% increase in death with cancer rate were found during long-term follow-up in the lesser resection arm. Because of missing data in the original publication, a re-analysis was performed showing that overall survival was not statistically significantly different between both treatment arms, but this correction was published only one year later [6]. 

In the years that followed, several retrospective studies reported outcomes comparing lobectomy with sublobar resection. Kraeve et al. reported the 10-year outcomes of patients in the Surveillance, Epidemiology and End-Results (SEER) database, and found that patients undergoing lobectomy had significantly better survival rates when compared to segmentectomy in tumours <3 cm (cT1N0, stage IA) [7]. However, Kaplan–Meier survival curves remained similar in the first 3 years of follow-up. The general advice remained that lobectomy was the preferred treatment of choice for NSCLC, but a role might be present for sublobar resections in smaller, stage IA NSCLC patients. Other retrospective studies have demonstrated similar survival rates and local recurrence rates in favour of lobectomy [8,9,10,11,12,13,14,15]. A problem is that these studies frequently lacked adjustment for preoperative risk factors; thus, given the retrospective nature of these studies, it is possible that a selection bias was present, i.e., fitter patients would receive a lobectomy whilst sublobar resections were reserved for patients in poor overall health, resulting in lower OS. This was also confirmed in the systematic review by De Zoysa et al., concluding that lobectomy should be performed for early-stage NSCLC in younger patients with acceptable cardiopulmonary reserve [16]. The advantage of a decreased complication rate does not outweigh the increased locoregional recurrence rate. On the other hand, in elderly patients, a sublobar resection may yield comparable survival rates to lobectomy.

A meta-analysis by Ijsseldijk et al. compared lobar resection with parenchymal sparing resections for pT1a NSCLC [17]. Five-year OS and disease-free survival (DFS) after segmentectomy were similar to lobectomy with a relative risk (RR) = 1.08 (95% CI: 0.99–1.18). In most comparisons, wedge resections were similar to segmentectomy or lobectomy. Thus, for T1a NSCLC, parenchymal-sparing surgery has similar outcomes to lobectomy; however, an important concern is the risk of nodal upstaging. Two randomised trials were initiated to obtain more evidence: JCOG0802/WJOG4607L in Japan, and CALBG 140503 in North America.

In the Cancer and Lymphoma Group B (CALBG 140503) trial, patients with a suspected or confirmed cT1aN0 peripheral NASCLC ≤ 2 cm were included. After confirmation of diagnosis and negative hilar and mediastinal LNs, patients were intraoperatively randomised to lobectomy or to sublobar resection, comprising segmentectomy and wedge resection [18,19]. Pure ground-glass opacities (GGO) were excluded. The primary endpoint was DFS with the following secondary endpoints: OS, pulmonary function and recurrence rates. In total, 357 patients were included in the lobectomy group and 340 patients in the sublobar group, of which 58.8% were wedge resections. There was no significant difference in 5-year DFS between 63.6% (sublobar resection) and 64.1% (lobar resection). For OS, non-inferiority of sublobar resection was confirmed with a one-sided *p* = 0.014. There was no significant difference in lung- and non-lung related deaths or disease recurrence in both arms. When comparing pulmonary functions at 6 months to the baseline function, there was a significant difference in median forced expiratory volume in one second (FEV1) change from baseline for lobectomy and sublobar resection (*p* = 0.0006). Change in forced vital capacity (FVC)% approached significance, but remained at *p* = 0.0712 in favour of sublobar resection. 

Sublobar resection was not inferior to lobectomy for the primary endpoint of DFS or the secondary endpoint of OS. Disease recurrence at 30% was also similar in both study arms. Even though differences in respiratory functions were observed, in favour of sublobar resection, the clinical significance is questionable. This trial confirms the non-inferiority of sublobar resection which still entails a 30% recurrence rate.

In the Japan Clinical Oncology Group and the West Japan Oncology Group trial (JCOG 0802/WJOG4607L), patients were included with a cT1a peripheral NSCLC or suspected nodule, a maximum tumour diameter of ≤2 cm and in the case of GGO, a consolidation-to-tumour ratio (CTR) >0.5 [20]. Patients were then randomised to lobectomy or segmentectomy (wedge excisions were not allowed). The primary endpoint of 5-year OS demonstrated a better outcome for segmentectomy of 94.3% vs. 91.1% (HR 0.663 (95% CI: 0.474–0.927) *p* = 0.0082) for superiority. Secondary endpoints however, showed similar 5-year relapse-free survival with segmentectomy at 88.0% and lobectomy at 87.9% (HR 0.998 (95% CI: 0.753–1.323) *p* = 0.9889). The recurrence pattern demonstrated a significantly higher proportion of local recurrences of 10.5% in the segmentectomy arm and 5.4% in the lobectomy group (*p* = 0.0018). However, the total number of lung cancer deaths were similar at 4.7% for segmentectomy versus 4.1% for lobectomy. The overall mortality was higher in the lobectomy group at 14.9% vs. 10.5%. However, this was primarily due to other deaths such as other malignancies at 5.6% (lobectomy) and 2.2% (segmentectomy) and non-malignant disease at 3.8% (lobectomy) vs. 2.7% (segmentectomy). This remains to be further explored.

NCT02011997 is an ongoing Chinese randomised control trial comparing complete Video-Assisted Thoracoscopic Surgery (cVATS) lobectomy to cVATS segmentectomy. An estimated 500 patients will be included involving a stage IA NSCLC with adenocarcinoma in situ or with microinvasion. A 5-year postoperative follow-up will be performed and recurrence-free survival has been chosen as the primary endpoint. The secondary endpoints are: 5-year survival rate, pulmonary function at 6 months follow-up, postoperative complications, and quality of life assessment. Patient enrolment commenced in December 2013; however, no results have been published yet.

With the new evidence provided, sublobar resection should be the new standard treatment modality in patients with peripheral small stage IA NSCLC tumours (≤2 cm) without lymph node metastases. There might be a place for tumours up to 3 cm in the outer third of the lung parenchyma; however, compelling evidence is lacking. Further subgroup analysis with the merging of data from recent randomised trials might contribute to outcome differences in the type of sublobar resection (wide wedge excision and anatomical segmentectomy), NSCLC histology, secondary cancers, and comorbidity. 
